# The Significance of Probiotics in Aquaculture: A Review of Research Trend and Latest Scientific Findings

**DOI:** 10.3390/antibiotics14030242

**Published:** 2025-02-27

**Authors:** Elshafia Ali Hamid Mohammed, Abdelhakam Esmaeil Mohamed Ahmed, Béla Kovács, Károly Pál

**Affiliations:** 1 Department of Animal Husbandry, Institute of Animal Science, Biotechnology and Nature Conservation, Faculty of Agricultural and Food Sciences and Environmental Management, University of Debrecen, 4032 Debrecen, Hungary; 2 Doctoral School of Animal Science, University of Debrecen, 4032 Debrecen, Hungary; 3 Agricultural Research Corporation, Integrated Pest Management Research Center, Wadmadani P.O. Box 126, Sudan; 4 Institute of Food Sciences, Faculty of Agricultural and Food Sciences and Environmental Management, University of Debrecen, 4032 Debrecen, Hungary; ahmed.abdelhakam@agr.unideb.hu (A.E.M.A.); kovacsb@agr.unideb.hu (B.K.); pal.karoly@agr.unideb.hu (K.P.)

**Keywords:** probiotics, aquaculture, antibiotics, disease resistance, growth performance, bibliometric analysis, VOSviewer

## Abstract

Millions of people around the world rely on aquaculture as a major source of food. In the recent few years, probiotics have gained considerable attention as an alternative agent to antibiotics. They have been shown to play an important role in improving aquaculture species through different mechanisms, mainly disease management, improving their growth performance, and improving water quality. Consequently, this review aimed to identify the key areas of research in the global literature about the influence of probiotics on aquaculture based on the selected keywords “aquaculture” AND “probiotics” AND “growth performance” AND “disease resistance” (APGD). The meta-data of the published literature were extracted from the Web of Science (WoS) database on 23 December 2024, and then the co-authors, countries, and keywords were analyzed and visualized using VOSviewer (v. 1.6.20). The search found a remarkable number of documents, which included 175 APGD documents. The results of the bibliometric analysis of the global literature reveal a substantial increase in the publication of APGD documents from 2019 to 2024. Asia, particularly China (32.3% of documents), has emerged as a leader of APGD publications, followed by Iran (8.67%), India (8.01%), Malaysia (7.5%), and Spain (7.5%), respectively. Keyword analysis revealed the prevalence of disease resistance (length = 1793), probiotics (1348), aquaculture (1169), and growth performance (913) as the most impactful keywords based on the WoS database. This could indicate that most of the APGD documents were focused on disease resistance and probiotics relationships. In addition, an extensive review of the recent literature showed that probiotics have demonstrated a remarkable efficacy in improving the growth performance, feed utilization efficiency, disease prevention, and water quality management in various aquaculture species under different aquaculture systems when used as feed or water additives for 30–90 days. It can be concluded that Asia is the lead continent in aquaculture probiotics research, with a significant increase in APGD documents in the last 5 years. Probiotics played a major role in improving aquatic species. This research aims to provide valuable insight into the use of probiotics in aquaculture and highlights the need for further research to fully understand their benefits and mechanisms of action.

## 1. Introduction

According to the Food and Agriculture Organization (FAO) [1], the aquaculture industry constitutes a substantial food source for millions of individuals globally. A review of the fishery yearbook [2] indicates that approximately 89% of the global total fish production in 2024 was utilized directly for human consumption. However, the presence of pathogens has been observed, which can negatively impact fish production. These pathogens are predominantly gram-negative bacteria, including *Aeromonas salmonicida*, *A. hydrophila*, *Vibrio harveyi*, *V. anguillarum*, *Flavobacterium psychrophilum*, *Pseudomonas fluorescens*, *Citrobacter freundii*, and *Yersinia ruckeri* [3]. Less frequently, gram positive bacteria such as *Streptococcus* spp. are also involved. The combined effect of these pathogens and unfavorable environmental factors is deleterious to productivity, resulting in significant economic losses for farmers. Poor fish management practices, including excessive stock densities, overfeeding, and water contamination, also contribute to the occurrence and spread of pathogens in aquaculture environments [4,5]. Antibiotics are frequently utilized in aquaculture as a means of combating diseases and enhancing fish performance. However, the continuous use of antibiotics in aquaculture has increased the selective natural pressure on the microbial community, leading to the emergence of antibiotic-resistant bacterial strains that can disseminate widely [6]. These resistant bacteria can spread rapidly, negatively impacting aquaculture production, fish consumers, and the surrounding environment [7,8,9]. In the context of these concerns, probiotics are being explored as an alternative technique that can be used as feed or water additives to promote environmental sustainability and the aquaculture industry [10].

As posited by Gismondo et al. [11], the term “probiotic” is derived from two Greek words, “pro” and “bios”, which together signify “for life”. However, the initial definition of the term probiotic was put forth by Parker [12], who defined a probiotic microorganism as one that contributes to intestinal microbial balance. As new findings have emerged, several alternate definitions of probiotics have been proposed. According to FAO and the World Health Organization (WHO) [13], probiotics are “live microorganisms which, when administered in adequate amounts, confer a health benefit to the host”. Probiotics have been found to be effective in a variety of aquatic environments, including freshwater [14], marine water, and brackish water [15]. The most commonly used probiotics are from the lactic acid bacteria (LAB), including the genera *Leuconostoc*, *Pediococcus*, *Lactococcus*, *Oenococcus*, and *Enterococcus* [16].

A substantial body of research has demonstrated the efficacy of the use of probiotics in a diverse range of fish and aquatic organisms such as African catfish (*Clarias gariepinus*), rainbow trout (*Oncorhynchus mykiss*), Nile tilapia (*Oreochromis niloticus*), European sea bass (*Dicentrarchus labrax*), rohu (*Labeo rohita*), snook (*Centropomus undecimalis*), and Red seabream (*Pagrus major*) [17,18,19]. Probiotics confer benefits to their hosts via a variety of mechanisms, including the enhancement of growth parameters [20,21,22,23,24] and the reduction of disease incidence [22,25]. Several factors can influence the effectiveness of probiotics, including the animal host, the strain of probiotic, the dosage, and the specific environmental conditions of the aquatic system. For instance, different strains of *Bacillus* have been shown to exhibit strain-specific benefits, with some strains providing significant growth rates and immune benefits while others do not [26]. This highlights the importance of selecting appropriate probiotic strains that are tailored to the specific needs of the aquaculture species being cultured.

The present review aims to provide a bibliometric analysis of the trends in the utilization of probiotics in aquaculture, with the objective of identifying research gaps in the existing literature concerning the interaction between probiotics and aquaculture under various conditions. Moreover, the specific objectives of the current study were as follows: (A) to identify the key areas of focus of research about the influence of probiotics in aquaculture based on the selected keywords (aquaculture, probiotics, growth parameters, and disease resistance); (B) to identify specific effects of probiotics on the growth performance and feed utilization of aquaculture species; (C) to identify the impact of probiotics on disease prevention, water quality (nitrogenous materials), and the survival of aquaculture species; and (D) to identify the impact of the interaction between different strains of probiotics and synbiotics in aquaculture.

## 2. Materials and Methods

### 2.1. Research Questions

- What are the key areas of research, research volume, geographical distribution of the literature, co-authoring counties, and leading publishers of the studies that focus on the influence of probiotics in aquaculture? This investigation was based on a selection of keywords, including “aquaculture”, “probiotics”, “growth performance”, and “disease resistance”;
- What are the specific effects of probiotics on fish growth performance, fish feed utilization, and the survival rate of aquaculture species?
- What is the impact of probiotics on disease prevention, water quality (nitrogenous materials), and survival in aquaculture species?
- What is the impact of the interaction between different strains of probiotics and synbiotics in aquaculture?

### 2.2. Searching and Exclusion Strategy

A thorough investigation was conducted by examining the worldwide literature in the Web of Science (WoS) database. WoS was selected because of its reputation as the most comprehensive and widely used database of literature in the area of reviews and bibliometric analyses. The search was conducted using a combination of keywords including “aquaculture”, “probiotics”, “growth performance”, and “disease resistance”, with a time frame extending from 2008 to 2024. All documents originally written in English were included. In addition, all open access research articles or book chapters were included; however, other types of documents were excluded. The search yielded a substantial number of documents, which was 175 in total.

### 2.3. Bibliometric Analysis

**Figure 1:**
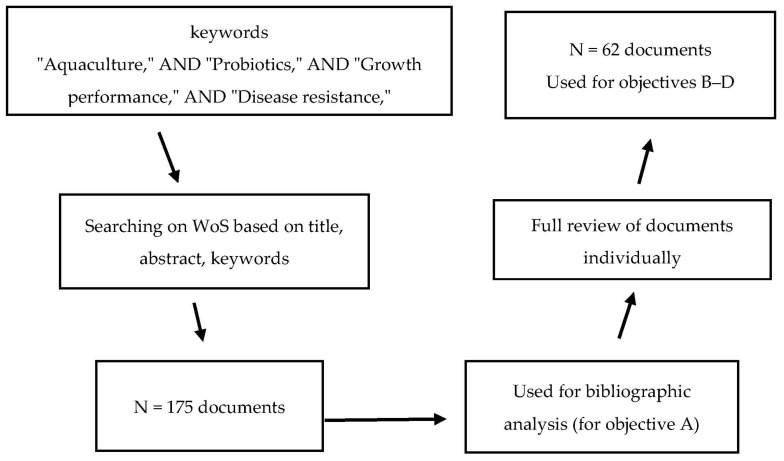
Workflow screening, extraction, and processing of the obtained metadata from Web of Science database (WoS) from 2008–2024.

The first objective of this study (objective A) was to perform a screening of WoS based on titles, abstracts, and keywords. The obtained metadata comprised 175 research articles or book chapters that contained at least one of the search keywords. These 175 documents were then selected for bibliometric analysis (Figure 1). The bibliometric information, including names, countries of affiliation, document titles, keywords, publication dates, and publishers, were exported. Subsequently, co-occurrence (all keywords, significant keywords to the research question), co-authors’ countries, and total links were analyzed according to the method described by Van Eck and Waltman [27] using VOSviewer (v. 1.6.20) (CWTS, Leiden, The Netherlands). The results were then visualized as clusters to identify possible research gaps and to highlight knowledge limitations with relation to the regions (countries) where the research studies were conducted.

### 2.4. Screening and Data Extraction

Regarding this study’s objectives, data including country, probiotic species/strain, aquatic animal host, growth parameters, feed utilization indications, situation of disease incidence, gut integrity and microbiome of animal host, level of ammonia and toxic material in water, and activity of digestive enzymes were extracted directly from the results of reviewed articles or by using Web Plot Digitizer (v. 5.0) (Ankit Rohatgi, Austin, TX, USA) if the data presented as figures. The extracted results were then summarized, arranged in tables, and discussed.

## 3. Results and Discussions

### 3.1. Situation of the Scientific Documents Based on WoS Search on Aquaculture, Probiotics, Growth Performance and Disease Resistance (APGD)

#### 3.1.1. Leading Countries on APGD Research

**Figure 2:**
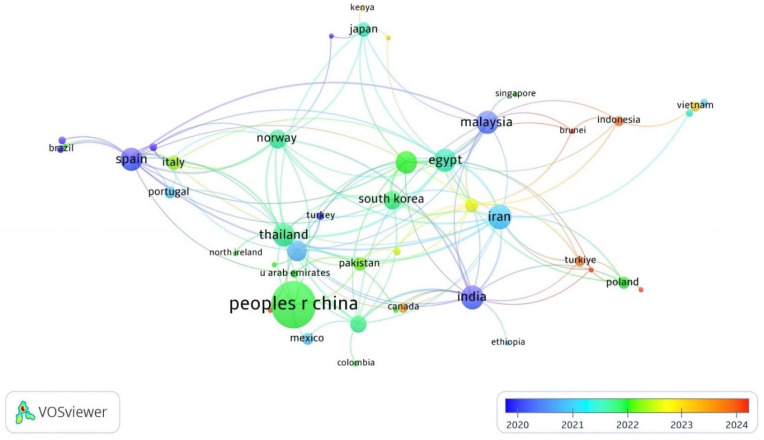
Network visualization of co-authoring countries of APGD documents based on Web of Science (WoS) database, 2008–2024. APGD: aquaculture, probiotics, growth performance, and disease resistance.

In total, 56 countries have published at least one research document on APGD based on the WoS database. In fact, 175 documents have been published by 57 countries. Asia was the leading continent for APGD-related documents, with co-authors of 85.1% of the documents published by the top 20 APGD countries (Table 1). China (nine total links) was the largest contributor with 56 APGD documents (Figure 2), representing around 32.3% of the total APGD documents published in the years 2008–2024 in the WoS database. In fact, China is the world’s largest producer of aquatic species, accounting for approximately 35% of the world’s fish and seafood production [28]. China’s aquaculture sector is characterized by its vast scale and diversity, producing more than 49,000,000 tons of fish in 2020. This diversity includes not only finfish but also mollusks and crustaceans, allowing for a wide range of products that cater to both domestic and international markets [28]. The top three contributors of APGD documents after China were Iran (15 documents), India (14 documents), and Malaysia (13 documents); the total links of these countries were 25, 22, and 17 for Iran, India, and Malaysia, respectively. The other leading countries with significant contributions to APGD documents were Spain, Thailand, Egypt, Bangladesh, the USA, Norway, Republic of Korea, Australia, Italy, Japan, Pakistan, Saudi Arabia, Poland, and Portugal, respectively (Table 1).

#### 3.1.2. Years of Publication and Publishing Entities

**Figure 3:**
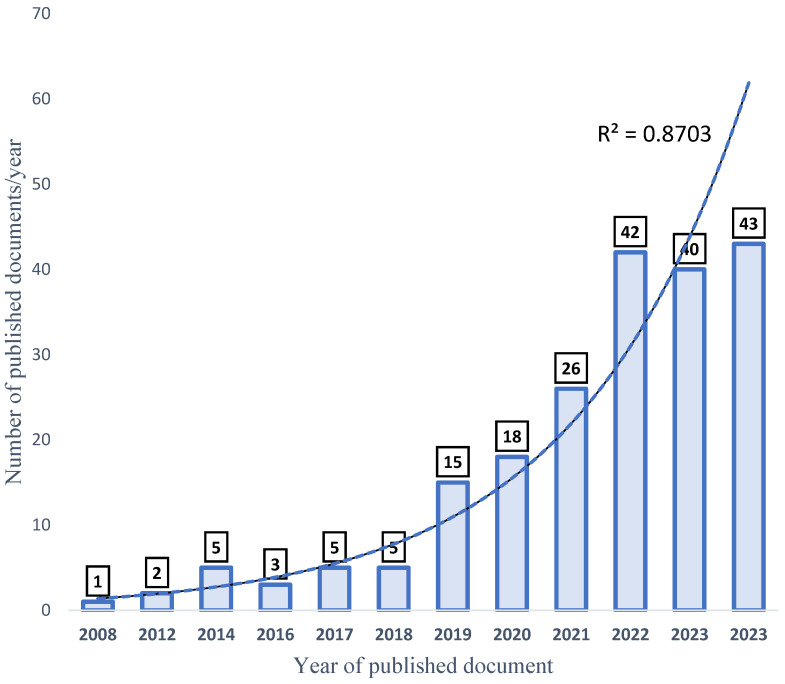
Progress of publication of APGD documents, based on Web of Science (WoS) database, between 2008 and 2024. APGD: aquaculture, probiotics, growth performance, and disease resistance.

The total amount of documents published about the selected keywords (aquaculture, probiotics, growth performance, and disease resistance (APGD)) in the date range from 2008 to 2024 was 175. However, in one decade (between 2008 and 2018), only 19 documents (1–5 documents/year) were published about APGD, representing 10.98% of the total published documents. Later, a high increase in the publication rate (%/year) occurred, and the progress was exponential, which is justified by the high R^2^ value (0.9) (Figure 3). In 2019, the number of published documents was 13 (7.5%), while 15 (8.7%), 24 (13.9%), 37 (21.4%), 30 (17.3%), and 37 (21.4%) documents were published in the years 2019, 2020, 2021, 2022, 2023, and 2024, respectively. This rapid increase in the publication of scientific papers signifies a rapid response to address the importance of aquaculture, the rapidly growing human population, and food security-related issues. Projections indicate that, by the year 2050, the global population will be more than 9 billion, with a projected 85% increase in food production [29].

**Figure 4:**
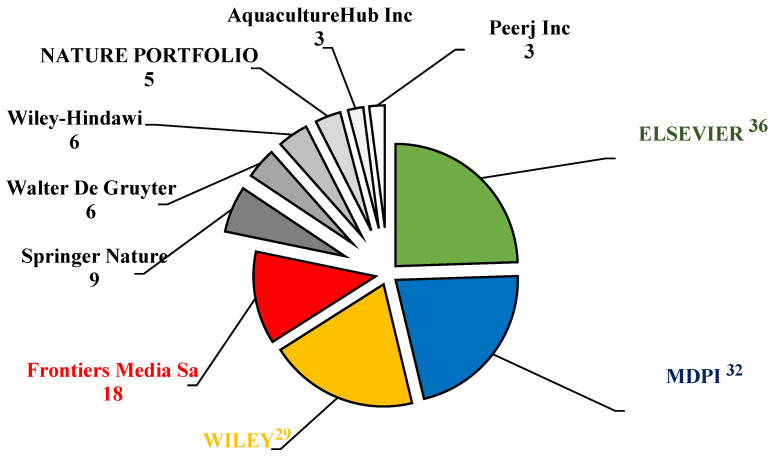
Leading publishers of the APGD documents based on Web of Science (WoS) database, 2008–2024. APGD: aquaculture, probiotics, growth performance, and disease resistance.

Elsevier and the Multidisciplinary Digital Publishing Institute (MDPI) were the most prominent academic publishers in aquaculture-related sciences. In this investigation, the top three publishers of APGD documents were Elsevier (36 documents), MDPI (32 documents), and Wily (30 documents) (Figure 4). These publishers played a vital role by publishing more than 58% of the total APGD documents published between 2008 and 2024. The dramatic increase in the research output by China has positioned it as a leading player in global academic publishing, including the publishing of studies related to aquaculture. China now exceeds the United States and any other nation in terms of its number of researchers and scientific publications, with a significant rise in the quality of these publications also being seen, as evidenced by their increasing number of citations in top-ranked journals [30]. MDPI has emerged as a major player in the publication of APGD documents and is particularly noted for its open access model, which allows for the rapid dissemination of research results. This is crucial in a field like aquaculture, where timely information can significantly impact practices and policies. For instance, a recent analysis highlighted a gradual increase in sustainable aquaculture publications, with a notable peak in 2023, indicating a growing interest and the need for accessible research in this area [31]. The open access nature of the MDPI journals ensures that research is readily available to practitioners and policymakers, facilitating the application of new findings in real-world settings.

### 3.2. Volume of Research on APGD Based on WoS Database

#### 3.2.1. Co-Occurrence of All Keywords

**Figure 5:**
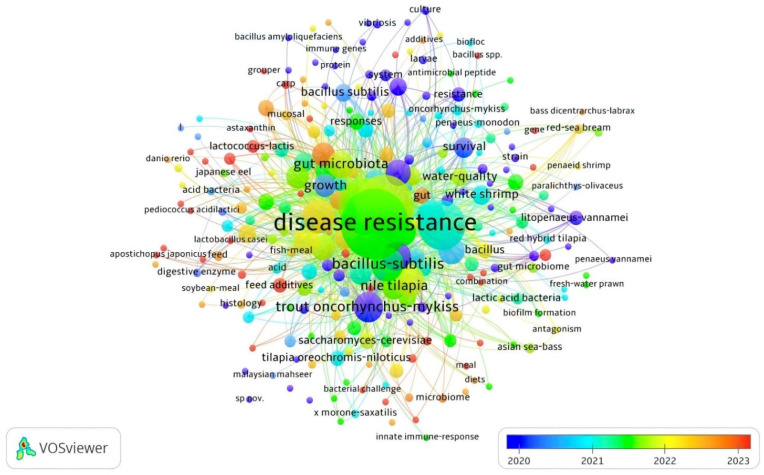
Network visualization of all keywords in APGD documents based on Web of Science (WoS) database, 2008–2024.

According to an analysis of co-occurrence of all keywords in the APGD documents from between the years 2008 and 2024, the total number of instances of the co-occurrence of all keywords was 939. However, the most significant keywords appearing in the APGD documents and their frequency of appearance (per time) were disease resistance (130), probiotics (99), aquaculture (88), growth-performance (67), *Bacillus subtilis* (35), and fish (36), with the total length strength recorded being 1793, 1348, 1169, 913, 491, and 482 for disease resistance, probiotics, aquaculture, growth performance, *Bacillus subtilis*, and fish, respectively (Table 2, Figure 5). This means that these keywords, within the past few years, have had central importance in aquaculture probiotics research. In fact, a higher total link strength of a keyword indicates a higher degree of connectivity of that word with other keywords and a higher likelihood that it appears with other keywords in the same document. The other significant keywords were performance, immunity, trout (*Oncorhynchus mykiss*), dietary supplementation, rainbow trout, immune response, lactic acid bacteria, bacteria, gut microbiota, innate immunity, growth performance, Nile tilapia, and probiotic, respectively (Table 2).

#### 3.2.2. Co-Occurrence of Author Keywords

**Figure 6:**
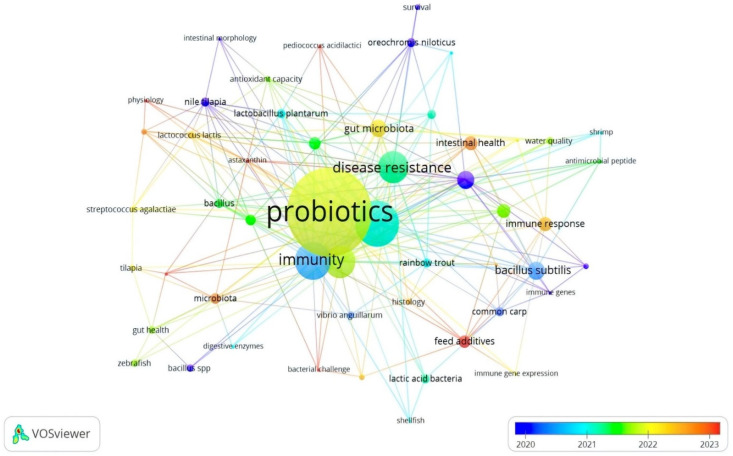
Network visualization of co-occurrence of author keywords in APGD documents based on Web of Science (WoS) database, 2008–2024. APGD: aquaculture, probiotics, growth performance, and disease resistance.

Similarly, the most significant author keywords were probiotics (62), aquaculture (30), immunity (24), probiotic (22), and growth performance (21). The total length strength of these keywords was 291, 154, 122, 109, and 108 for probiotics, aquaculture, immunity, probiotic, and growth performance, respectively (Table 3). The other keywords were disease resistance, growth, *bacillus subtilis*, *Litopenaeus vannamei*, gut microbiota, immune response, intestinal microbiota, intestinal health, gene expression, feed additives, *Aeromonas hydrophila*, and microbiota (Table 3, Figure 6). Based on the obtained results (Table 2), the keywords disease resistance (1793), probiotics (1348), aquaculture (1169), growth-performance (913), *Bacillus subtilis* (491), fish (482), and performance (456) yielded the highest length strengths, which can indicate that, between 2008 and 2024, the APGD documents focused on improving growth and disease resistance through the implementation of feed-based probiotics. Numerous studies have shown that overcrowding in intensive aquaculture systems can lead to stress in fish, making them more susceptible to infections and allowing pathogens to be more aggressive [32,33,34]. The stress induced by high stocking densities can compromise the immune systems of fish, increasing their vulnerability to diseases such as streptococcosis and viral infections [35]. However, probiotics are implemented for sustainable aquaculture purposes and to enhance growth parameters [20,21,22,36,37] or decrease the likelihood of disease outbreaks [22,25,38].

### 3.3. Impact of Probiotics on Growth Parameters

#### 3.3.1. Growth Indices

The parameters that are commonly used to evaluate animal growth performance after treatment with probiotics-based feed include the final body weight (FBW, g), survival rate (%), feed conversion ratio (FCR, g/g), and specific growth rate (SGR, %). The FCR value is calculated using the formula FCR = F/ (Wf − Wi), where F represents the feed intake, Wf is the final body weight, and Wi is the initial wet body weight. The SGR is determined by the formula SGR = (lnWf − lnWi)/t × 100, where Wf is the final body weight, Wi is the initial wet body weight (g), and t is the time (days).

Enhanced growth-related parameters are reflected in faster harvesting times and improved production efficiency, which is crucial for meeting the rising global demand for seafood [39]. Numerous studies have discussed the positive correlation between probiotics and the growth performance of different aquatic animals. For instance, a report from China stated that the growth performance of sea cucumber (*Apostichopus japonicus*) can significantly be improved when the animal receives a diet supplemented with the probiotic *Sporosarcina aquimarina* MS4 at 10^7^ cfu/g after 60 days [40]. In addition, the FCR, FBW, SGR (%/day), and weight gain (%) of common carp (*Cyprinus carpio*) can be significantly improved when the fish receive a diet supplemented with probiotic *Pediococcus pentosaceus* for 45 days [41]. Similarly, a report from China indicated that the probiotics *Limosilactobacillus reuteri* FS3052, *Lacticaseibacillus rhamnosus* FS3051, and *Bacillus subtilis* natto NTU-18 significantly improved the growth indices of Grey mullet (*Mugil cephalus*) when the fish (1.07 ± 0.07 g) were fed a diet supplemented with probiotics for 28 days [42].

In the last few years, extensive research studies have been performed on a wide range of aquaculture species, especially fish and shrimp, to evaluate the impact of probiotic-based diets on their growth parameters and feed efficiency (Table 4). Many research studies have confirmed the positive correlation between the use of probiotic and fish growth indices; however, the exact mechanisms by which probiotics improve growth parameters are still not fully understood.

#### 3.3.2. Mechanism of Action

According to Eliopoulos et al. [43] and Yang et al. [44], probiotics play an important role in improving feed palatability through the fermentation process. The improvement of feed palatability by probiotics can be attributed to their ability to modify the sensory characteristics of feed. For instance, the fermentation process carried out by certain probiotic strains can produce metabolites that enhance the aroma of feed, which plays an important role in sensory perception, making it more attractive to animals. Yang et al. [44] demonstrated that the palatability of traditional Chinese herbs could be effectively enhanced by probiotic fermentation, enhancing their flavor and taste. Furthermore, a recent study showed that the flavor and palatability of plants could be altered after their fermentation by probiotics, which results in the subsequent stimulation of the animals’ appetite by the formation of aroma and flavor compounds [45].

Lactic acid bacteria, particularly *Lactobacillus lactis*, have been shown to play a critical function in the digestion of the macro-nutrients of feedstuffs fed to fish, which has been associated with enhanced hydrolysis and increased nutrient utilization in several fish species [46]. Furthermore, probiotic-supplemented diets have been shown to have the capability to improve fish performance through enhancing the activity of digestive enzymes and improving food absorbability [17,47,48]. For example, Zhang et al. [49] noticed that the digestive enzymes (lipase, amylase, and trypsin) of farmed tilapia (*Oreochromis niloticus*) were efficiently improved after 45 days of the fish receiving diets supplemented with the probiotic *Clostridium butyricum* at concentrations of 1.5 × 10^11^ CFU/kg. In addition, the dietary inclusion of *Lactobacillus* spp. and *L. pentosus* at concentrations of 10^7^ and 5 × 10^8^ CFU per g improved several types of digestive enzymes of white shrimp (*Litopenaeus vannamei*) [50,51]. Furthermore, Tang et al. [52] noticed that the activity of protease and amylases enzymes of *Siniperca chuatsi* significantly improved when fish received a diet supplemented with the probiotic *Bacillus velezensis GY65*.

**Table 4:** Specific effects of probiotics on fish growth performance and feed utilization based on recent studies.

Country	Host × Probiotic	Experimental Condition	Main Outcomes	Reference
China	▪ Grey mullet (*Mugil cephalus*) ▪ *Lacticaseibacillus rhamnosus* FS3051 (LA), *Limosilactobacillus reuteri* FS3052 (LI), and *Bacillus subtilis* natto NTU-18 (BS)	▪ 144 fish (1.07 ± 0.07 g) were distributed in 4 tanks (36 fish/tank) in RAS ▪ T1: received control diet only, T2–T4: received diet supplemented with LA, LI, and BS respectively ▪ Supplementation: feed additive ▪ Experiment period: 28 days, feeding rate: 2/day ▪ Water volume was 30 L/tank, temperature of 24–26 °C.	▪ FBW (g): LA ↑, LI ↑, BS ↑ ▪ FCR (g): LA ↑, LI ↑, BS ↑ ▪ SGR (% day^−1^): LA ↑, LI ↑, BS ▪ FL (mm): LA ↑, LI ↑, BS ↑ ▪ SGR (%): LA ↑, LI ↑, BS ↑ ▪ LGR (%): LA ↑, LI ↑, BS ↑	[42]
Mexico	▪ *Lactococcus lactis* PH3-05 ▪ tropical gar adults (*Atractosteus tropicus*)	▪ Fish larvae (0.02 ± 0.00 g) were distributed in 4 treatments, 3 diets were incorporated with *L. lactis* PH3-05 at 10^4^, 10^6^, and 10^8^ CFU/g, and a control diet without probiotics (CD) ▪ Supplementation: feed additive ▪ Temp.: 27.1 ± 0.1 °C, pH: 7.3 ± 0.5, DO: oxygen: 5.5 ± 0.2 mg/L ▪ Feeding rate: 6/day	▪ FBW (g): (CD) 0.031 ± 0.002 b, (10^4^): 0.034 ± 0.0004 b, (10^6^): 0.040 ± 0.00 a, (10^8^): 0.041 ± 0.00 a ▪ SGR (% d^−1^): (CD): 1.53 ± 0.07 b, (10^4^): 1.64 ± 0.07 b, (10^6^): 2.75 ± 0.15 a, (10^8^): 2.83 ± 0.003 a	[53]
China	▪ *Salmo trutta* ▪ *Saccharomyces cerevisiae* (SC), *lactic acid bacteria* (LAB), *Bacillus licheniformis* (BL)	▪ 360 *S. trutta* (84.57 ± 1.83 g) were distributed in 4 groups (cages) with triplicate (30 fish/cage). G1, received control diet (CD), while G2 to G3 received control diet supplemented with SC, LAB, and BL respectively ▪ Supplementation: feed additive ▪ Feeding rate: 2 time/day ▪ DO: 6.2~7.3 mg/L, temperature: 10~12 °C, water level: 0.6 m	▪ BW (g): (CD) ↓, LAB ↑, SC ↑, BL ↑. ▪ FL (cm): (CD) ↓, LAB ↑, SC ↓, BL ↑. ▪ Survival (%): (CD) ↓, LAB ↑, SC ↑, BL ↑ ▪ WG (%): (CD) ↓, LAB ↑, SC ↑, BL ↑ ▪ AGR (%): (CD) ↓, LAB ↑, SC ↑, BL ↑	[54]
China	▪ *Siniperca chuatsi* ▪ *Bacillus velezensis GY65*	▪ Initial body weight: 29.4 g ± 0.2 g ▪ Duration: 8 weeks ▪ Supplementation: feed additive	▪ WGR (%): ↑	[52]
Egypt	▪ Nile tilapia (*Oreochromis niloticus*) ▪ Aqua Star^®^ (*Bacillus* sp., *Pediococcus* spp., *Enterococcus* spp.)	▪ Fish (46 g) were distributed in 4 groups, G1: probiotic free, G2–G4 received diet supplemented with 0.0010, 0.0015, and 0.0020 g m^−3^ day^−1^, respectively ▪ Supplementation: feed additive ▪ Feeding: 3 times/day	▪ FBW (g): G1 ↓, G2 ↑, G3 ↑, G4 ↑ ▪ WG (g): G1, G2 ↑, G3 ↑, G4 ↑ ▪ WGR (%): G1 ↑, G2 ↑, G3 ↑, G4 ↑ ▪ FCR: G1 ↑, G2 ↓, G3 ↓, G4 ↓ ▪ NH_3_ (ppm): G1 ↑, G2 ↓, G3 ↓, G4 ↓ ▪ Survival (%): G1 ↓, G2 ↑, G3 ↑, G4 ↑	[55]
China	▪ Hybrid grouper (*Epinephelus lanceolatus*♂ × *Epinephelus fuscoguttatus*♀) ▪ *Bacillus subtilis* strains, 6-3-1, BS, and HAINUP40	▪ The experiment had 4 groups. BS diet (control feed + BS strain), a 6-3-1 diet (control + 6-3-1 strain), a HAINUP40 diet (control + HAINUP40 strain), and a control (+only PBS) ▪ Supplementation: feed additive ▪ Duration: 42 days	▪ Control: FBW ↓, WGR ↓ (%), FCR ↑, FER ↓ ▪ BS: FBW ↓, WGR (%) ↓, FCR ↑, FER ↑ ▪ 6-3-1 strain: WGR (%) ↑, FCR ↓, FER ↑ ▪ HAINUP40 strain: WGR (%) ↑, FCR ↓, FER ↑	[26]
Indonesia	▪ tilapia (*Oreochomis niloticus*) ▪ *Lactococcus garvieae*, *Priestia megaterium*, *Bacterium* spp	▪ Fish (14.39 ± 1.02 g) were distributed into 4 groups. G1: received probiotics free diet, while G2–G4 received diet supplemented with *L. garvieae*, *P. megaterium*, *Bacterium* spp., respectively. ▪ Supplementation: feed additive ▪ Feeding: 2/day ▪ Duration: 60 days	▪ WG (%): G1 ↓, G2 ↑, G3 ↑, G4 ↑ ▪ SGR (%/day): G1, G2 ↑, G3 ↑, G4 ↑ ▪ FCR: G1 ↑, G2 ↓, G3 ↓, G4 ↓	[56]
Spain	▪ gilthead seabream (*Sparus aurata* L.) ▪ *Halobacterium salinarum*	▪ Fish (8 ± 1.07 g) were distributed into 3 groups. G1 received control diet only while G2 and G3 received diet supplemented with lyophilized *H. salinarum* at: 0.05% and 0.1% respectively. ▪ Supplementation: feed additive	▪ FBW (%): G1 ↓, G2 ↓, G3 ↑ ▪ WG (g): G1 ↓, G2 ↓, G3 ↑ ▪ SGR (%/day): G1 ↓, G2 ↑, G3 ↑	[57]
Pakistan	▪ Nile tilapia (*Oreochromis niloticus*) ▪ *Lactobacillus rhamnosus*	▪ Fish (20 ± 2.0 g) were distributed into 5 groups and received different proportions of *L. rhamnosus* G1: 0 *L. rhamnosus*, G2: 0.5 × 10^10^ CFU/kg G3: 1 × 10^10^ CFU/kg, G4: 1.5 × 10^10^ CFU/kg, G5: 2 × 10^10^ CFU/kg Supplementation: feed additive ▪ Duration: 6 weeks	▪ BW (%): G ↓, G2 ↑, G3 ↑, G4 ↑, G5 ↑ ▪ WG (g): G ↓, G2 ↑, G3 ↑, G4 ↑, G5 ↑ ▪ SGR: G ↑, G2 ↑, G3 ↑, G4 ↑, G5 ↑ ▪ FCR: G1 ↓, G2 ↓, G3 ↓, G4 ↓, G5 ↓ ▪ LG: G ↓, G2 ↑, G3 ↑, G4 ↑, G5 ↑ ▪ PER: G ↓, G2 ↑, G3 ↑, G4 ↑, G5 ↑	[58]
China	▪ Nile tilapia (*Oreochromis niloticus*) ▪ *Lactobacillus reuteri*	▪ Fish (9.01 ± 0.11 g) were fed with a basal diet supplemented with 0 (control), 10^9^, 10^10^, or 10^11^ CFU/g of *L. reuteri* per kg. Temperature: 29 ± 1 °C, pH: 7.4 ± 0.7, ammonium: <0.50 mg/L, and nitrite: <0.05 mg/L. ▪ Supplementation: feed additive ▪ Duration: 8 weeks	▪ WG (g): 0, 10^9^ ↑, 10^10^ ↑, 10^11^ ↑ ▪ SGR (%/day): 0, 10^9^ ↑, 10^10^ ↑, 10^11^ ↑	[59]
Iran	▪ zebrafish (*Danio rerio*) ▪ *Lactobacillus casei*	▪ Fish (0.15 ± 0.01) were fed with control diet only (G1) or three levels of probiotic *L. casei* (10^5^, 10^6^, and 10^7^ CFU/g (G2, G3 and G4 respectively) ▪ Supplementation: feed additive ▪ Duration: 6 weeks	▪ Gene expression of growth-related genes (GH and IGF-1) were higher in probiotic groups. Similarly, FBW, WG, WGR were the highest with the high probiotic concentrations	[60]
China	▪ channel catfish (*Ictalurus punctatus*) ▪ *Saccharomyces cerevisiae*	▪ Fish (24.49 ± 0.11 g) were distributed into 2 groups. Control (basal diet) and SCC group (basal diet with 2% S. cerevisiae) ▪ Supplementation: feed additive ▪ Duration: 12 weeks	▪ FBW (g): control ↑, SCC ↑ ▪ WG (%): control ↑, SCC ↑ ▪ FCR: control ↑, SCC ↑	[61]
Egypt	▪ tilapia, (*Oreochromis niloticus*) ▪ *Lactobacillus plantarum*	▪ Fish (15.2 ± 0.6 g) were distributed into 4 groups as follows: T1 = a basal control diet, T2 = a basal + *L. plantarum*, T3 = a basal diet + 1.0 g whey protein concentrate (WCP)/kg diet and T4, T5 and T6 = basal diets + *L. plantarum* at + 1.0, 2.0 and 3.0 g of WCP/kg respectively ▪ Supplementation: feed additive ▪ Duration: 60 days	▪ FBW (g): T1 ↓, (T2–T6) ↑ ▪ WG (g): T1 ↓, (T2–T6) ↑ ▪ WG (%): T1 ↓, (T2–T6) ↑ ▪ SGR (%/day): T1 ↓, (T2–T6) ↑ ▪ FCR: (T1–T6) ns	[62]

AGR: average growth rate, FBW: final body weight, FCR: feed conversion ratio, SGR: significant growth rate, LG: body length gains, FL: final body length, FER: feed efficiency ratio, LGR: length gain rate, WGR: weight gain rate, PER: protein efficiency ratio. ↑: increase, ↓: decrease. Different letters mean significant differences. ns: non-significant.

### 3.4. Impact of Probiotic on Disease Resistance, Immunity and Water Quality

In addition to enhancing growth performance, the integration of probiotics in aquaculture has gained considerable attention for its ability to enhance disease resistance, modulate the immune system, improve gut health, and enhance microbial diversity within the guts of diverse fish and shrimp species. Thus, probiotics foster overall health in aquatic animals and contribute to the sustainability of aquaculture. In a previous study, Zokaeifar et al. [63] examined the impact of the probiotic *Bacillus subtilis* strains on juvenile white shrimp (*Litopenaeus vannamei*). They noticed that the cumulative mortality rate (%) of the shrimp in the control group following a challenge with *Vibrio harveyi* was 63.3%, while the probiotic groups exhibited significantly lower mortality rates, ranging from 20.0% to 33.3%.

A pivotal mechanism through which probiotics impart their salutary effects is by means of enhancing the expression of immune-related genes. For instance, the supplementation of *Bacillus cereus* in pengze crucian carp (*Carassius auratus*) has been shown to activate both specific and non-specific immune responses, thereby increasing their resistance to pathogens [64]. Furthermore, the administration of *Lactobacillus plantarum* has been associated with an enhancement in resistance against *Aeromonas sobria* in Nile tilapia (*Oreochromis niloticus*) [62] and *A. hydrophila* in Caspian whitefish (*Rutilus frisii kutum*) [65], suggesting a clear correlation between probiotic supplementation and enhanced disease resistance. A similar result has been recorded between the probiotic *Bacillus clausii* and increased lysozyme activity and complement C3 in grouper (*Epinephelus coioides*) following a 60-day period of feeding on a supplemented diet [66]. In recent years, numerous studies have examined the positive correlation between the administration of probiotics in aquatic animals and a reduction in pathogens, particularly *Staphylococcus*, *Mycoplasma*, and *Weissella* [67], *Mycoplasma* and *Rhodobacter* [42], and *Vibrio harveyi* [26] (Table 5).

Probiotic bacteria have been shown to have considerable beneficial effects on aquatic environments [68,69,70]. The concentrations of NO_3_⁻ (nitrate), NO_2_⁻ (nitrite), and ammonia (NH_4_^+^) are the main water quality parameters which effect fish performance and should be monitored regularly. These compounds are toxic to fish and can accumulate in aquatic ecosystems due to the decomposition of organic matter. Probiotics play a key role in reducing the concentrations of harmful substances in aquaculture water. Probiotic bacteria, particularly strains that belong to the genus *Bacillus*, have demonstrated the ability to assimilate nitrogen compounds and break down organic matter, thereby lowering the ammonia levels in water. It is believed that probiotic bacteria such as *Clostridium butyricum* can increase the number of denitrifying bacteria that can degrade NH_4_^+^ and NO_3_^−^ in water, resulting in a large amount of NH4-N and NO3-N being degraded into substances that are harmless to fish [71]. This process not only improves water quality but also creates a healthier environment for fish growth and development. Recently, Liu et al. [72] noticed that a mixture of probiotics (*Lactobacillus plantarum* and *Pediococcus pentosaceus*) significantly reduced the concentrations of ammonia and nitrite in the aquatic environment of Tilapia (*Oreochromis niloticus*) when the fish received a diet supplemented with *C. butyricum* at 3.0 × 10^10^, 1.5 × 10^11^, and 3.0 × 10^11^ CFU/kg [49].

**Table 5:** Impact of probiotics on disease resistance and immune-related parameters of some aquatic species.

Country	Host × Probiotic	Experimental Condition	Main Outcomes	Reference
China	▪ Grey mullet (*Mugil cephalus*) ▪ *Lacticaseibacillus rhamnosus* FS3051 (LA), *Limosilactobacillus reuteri* FS3052 (LI), and *Bacillus subtilis* natto NTU-18 (BS)	▪ 144 fish (1.07 ± 0.07 g) were distributed in 4 tanks (36 fish/tank) in RAS ▪ T1: received control diet only, T2-T4: received diet supplemented with LA, LI, and BS respectively ▪ Duration: 28 days, feeding: 2/day ▪ Supplementation: feed additive ▪ Water volume was 30 L/tank, temperature of 24–26 °C. ▪ 3 tanks with 10 fish for challenge trial, 1 tank with 6 fish for trials of gene expression, and gut microbiota	▪ LA: induced IL-8, IL-1β, TNF-α, IFN-γ, and MHCI ▪ BS: increased IL-8, MHCI, TLR2, IFN-γ ▪ LA: altered the gut microbiota, enriching *Lactobacillus* spp. and reduced *Mycoplasma* and *Rhodobacter*	[42]
Iran	▪ Caspian whitefish (*Rutilus frisii kutum*) ▪ *Pediococcus acidilactici* as a commercial probiotic (CP) and *Pediococcus pentosaceus* as host-associated probiotic (HAP)	▪ Three hundred whitefish fry (1.15 ± 0.03 g) were randomly divided into five treatments in triplicate ▪ Group 1 received control diet while G2 to G5 received (CP) at 6 × 10^8^ CFU g^−1^, (HAP) at 10^6^ (HAP1), 10^7^ (HAP2) and 10^8^ (HAP2) CFU g^−1^ respectively. ▪ Supplementation: feed additive ▪ Temperature: 22.1 ± 0.2 °C, DO: 6.89 ± 0.2 mg/L, pH: 7.6–7.9 ▪ Duration: 8 weeks	▪ lysozyme activity: CP ↑ and HAP ↑, no significant effect (ne)between both probiotics ▪ *A. hydrophila*: CP ↓, HAP1 ↓, HAP2 ↓, HAP3 ne ▪ HAP: LAB ↑ ▪ HAP and CP have no effect of total number of gut bacteria	[65]
China	▪ White leg shrimp (*Penaeus vannamei*) ▪ * Lactobacillus plantarum* Ep-M17	▪ 600 shrimp (4.5–5.5 cm) were distributed in 6 aquariums (400 L). 100 shrimp/aquarium. ▪ Feeding: 3 times/day ▪ Supplementation: feed additive ▪ Duration: 4 weeks	▪ Immunodigestive enzymes ↑ ▪ *V. parahaemolyticus* ↓ ▪ Immune response-related signals ↑ ▪ β-alanine ↑ ▪ histidine ↑ ▪ enhance shrimp disease resistance	[73]
China	▪ Hybrid grouper (*Epinephelus fuscoguttatus*♀ × *Epinephelus lanceolatus*♂) ▪ *Bacillus subtilis* strains (BS, HAINUP40, 6-3-1)	▪ The experiment had 4 groups. BS diet (basal feed with BS strain), a 6-3-1 diet (control + 6-3-1 strain), a HAINUP40 diet (control + HAINUP40 strain), and a control group (+PBS only) ▪ Supplementation: feed additive ▪ Duration: 42 days	▪ Control: Survival against *Vibrio harveyi *↓ ▪ BS: *Vibrio harveyi *↑ ▪ 6-3-1 strain: *Vibrio harveyi *↑ ▪ HAINUP40 strain: *Vibrio harveyi* ↓	[26]
China	▪ Turbot (*Scophthalmus maximus*) ▪ *Bacillus velezensis* T20 (T20)	▪ Fish (12.08 ± 0.76 g) were distributed into 2 groups: Control group received probiotic free diet and T20 received diet supplemented with *Bacillus velezensis* ▪ Supplementation: feed additive ▪ Feeding: 2 times/day ▪ Temperature: 17–20 °C, DO: 6–8 mg/L, salinity 27–29%, ammonia nitrogen NH_4_^+^-N < 0.3 mg/L, pH 7–8. ▪ Duration: 8 weeks	▪ T20: significantly reduced mortality caused by y *Edwardsiella tarda* ▪ Gut microbiota: *Bacillus* ↑, *Mycoplasma* ↓, *Staphylococcus* ↓, *Weissella* ↓	[67]
China	▪ whiteleg shrimp (*Litopenaeus vannamei*) ▪ lemon peel fermented with *Lactobacillus plantarum* (FLP)	▪ Shrimp (2.37 ± 0.01) were distributed into 4 groups. Group (1) received FLP free diet while other 3 groups received 1, 2, and 3% FLP respectively. ▪ Supplementation: feed additive ▪ Duration: 56 days	▪ Hemocyte count: 0 ↓, 1% ↑, 2% ↑, 3% ↓ ▪ Resistance against *Vibrio alginolyticus*: 0% ↓, 1% ↓, 2% ↑, 3% ↑	[74]
Bangladesh	▪ rohu (*Labeo rohita*) ▪ combination of *Bacillus subtilis* and *Lactobacillus* spp.	▪ larvae (0.003 g) were assigned into 3, group 1 received probiotic-free diet while other 2 groups received 0.5 and 1.0 mL/L of probiotics ▪ Supplementation: water additive ▪ Duration: 90 days	▪ Cellular abnormality due to *A. veronii* infection: ↑ ▪ goblet cell, and enterocytes of the gut were better in probiotic groups	[75]
China	▪ Chinese perch (*Siniperca chuatsi*) ▪ *Bacillus subtilis* 1-C-7	▪ Fish (30.3 ± 1.7) were distributed into 4 groups. G1: 0 cfu/g diet G2: 0.85 × 10^8^ cfu/g diet G3: 0.95 × 10^9^ cfu/g diet G4: 0.91 × 10^10^ cfu/g diet Supplementation: feed additive ▪ Duration: 10 weeks	▪ Survival against *A. hydrophila*: G1, G2 ↑, G3 ↑, G4 ↑ ▪ ALT: G4 ↑ ▪ AST: G4 ↑ ▪ Abundance of Tenericutes and Bacteroides: G2 ↑, G3 ↑, G4 ↑	[76]
China	▪ Zebrafish (*Danio rerio*) ▪ *Paenibacillus ehimensis* NPUST1	▪ Fish (0.61 ± 0.04 g) were distributed into 3 groups. G1: received control diet only while G2 and G3 received control diet supplemented with *P. ehimensis* NPUST1 at doses of 10^6^ and 10^7^ CFU/g respectively. ▪ Supplementation: feed additive ▪ Duration: 8 weeks.	▪ Survival against *A. hydrophila*: ▪ IL-6, (IL)-1β, IL-15, tumor necrosis factor-α were highly expressed in probiotic related groups (G2 and G3).	[77]
China	▪ Yellow catfish (*Pelteobagrus fulvidraco*) ▪ *Bacillus amyloliquefaciens*	▪ catfish (21 ± 1.2 g) were fed with control diet only (G1) or with 10^6^ cfu/g of *B. amyloliquefaciens* (G2) ▪ Duration: 4 weeks ▪ Supplementation: feed additive	▪ High resistance of fish fed *B. amyloliquefaciens* against *Edwardsiella ictaluri* and *Aeromonas veronii*.	[78]
México	▪ tilapia (*Oreochromis niloticus*) ▪ Yeast probiotic ▪ *Rhodoturula glutinis*	▪ Fish (7.02 ± 0.04 g) were randomly distributed into 3 groups. (control), Biofloc culture, and Biofloc with the addition of the *R. glutinis* 1 × 10^6^ CFU g^−1^ ▪ Supplementation: water additive ▪ Duration: 12 weeks	▪ Expression of immunity related genes such as *tnfa*, *tgfb*, *hsp70*, and *gpx* were significantly higher in probiotic group in Biofloc system	[79]
China	▪ tilapia (*Oreochromis niloticus*) ▪ *Clostridium butyricum*	▪ Fish received control diet only (G1) or *C. butyricum* at 3.0 × 10^10^, 1.5 × 10^11^ and 3.0 × 10^11^ CFU/kg for G2, G3 and G4 respectively ▪ Feeding: 2/day ▪ Supplementation: feed additive ▪ Duration: 90 days	▪ G1: NH_4_^+^ ↑, NO_3_^−^ ↑ ▪ G2: NH_4_^+^ ↓, NO_3_^−^ ↓ ▪ G3: NH_4_^+^ ↓, NO_3_^−^ ↓ ▪ G14: NH_4_^+^ ↓, NO_3_^−^ ↓	[49]
China	▪ *Pelteobagrus vachelli* ▪ *Rhodopseudomonas palustris*	▪ Fish (30 ± 5 g) were distributed into 4 groups. CK: the control group of water and commercial fish diet; WR: water and *R. palustris*; EO: effluent without *R. palustris*; and ER: effluent containing *R. palustris* ▪ Supplementation: water additive	▪ CK: NH_4_^+^ ↑ ▪ WR: NH_4_^+^ ↓ ▪ EO: NH_4_^+^ ↑ ▪ ER: NH_4_^+^ ↓	[72]

ALT: serum alanine aminotransferase, AST: aspartate aminotransferase, IL-8, IL-1β, TNF-α, IFN-γ: immune-related genes. ↑: increase, ↓: decrease.

### 3.5. Multi-Strain Probiotics and Synbiotics in Aquaculture

#### 3.5.1. Effect of Multi-Strain Probiotics

The utilization of multi-strain probiotics in aquaculture has garnered considerable attention due to their demonstrated capacity to enhance growth performance, fortify immune responses, and augment disease resistance in diverse aquatic species (Table 6). Multi-strain probiotics, which consist of a combination of different probiotic strains, can provide a synergistic effect that enhances the overall health and resilience of fish and shrimp against pathogens. For instance, the inclusion of multi-species probiotics has been shown to enhance the innate immune responses of whiteleg shrimp (*Litopenaeus vannamei*), leading to improved survival rates when they are challenged with pathogens such as *Vibrio parahaemolyticus* [80]. In a similar manner, dietary supplementation with a combination of *Bacillus subtilis* and *Lactobacillus plantarum* strains has been demonstrated to considerably enhance the immune parameters and disease resistance of Nile tilapia (*O. niloticus*) against *Aeromonas hydrophila* [62,81]. Furthermore, multi-strain probiotics have been associated with enhanced growth parameters in aquaculture species. Studies have shown that the use of multi-strain probiotic formulations can lead to better feed conversion ratios and increased weight gain in fish and shrimp [82]. For instance, a combination of *Bacillus subtilis* and *Lactobacillus casei* has been demonstrated to enhance growth performance in catfish (*Clarias gariepinus*) [83]. The synergistic effects of multiple strains have been demonstrated to optimize nutrient utilization and improve gut health, both of which are critical to growth and overall health in aquaculture [84].

#### 3.5.2. Synergistic Effect of Probiotics and Prebiotics

The combination of probiotics and prebiotics in synbiotics has been shown to have a synergistic effect [85]. Such a combination could improve the survival of probiotic organisms because, in the absence of its food source, a probiotic would face significant challenges in surviving in the digestive system. This is due to their inability to tolerate oxygen and low-pH and low-temperature conditions [86]. Synbiotics are therefore used to enhance growth and stimulate immune responses in the host [87].

A study by Saba et al. [88] reported that synbiotics exhibited superior performance compared to single-strain and multi-strain probiotics. The improved efficacy of synbiotics over single-strain and multi-strain probiotics can be attributed to the synergistic effect that occurs when the combined action of probiotics and prebiotics produces health benefits greater than the sum of their individual effects [89]. This synergistic effect has the potential to enhance digestive enzymes, health, and immune function [90]. Synbiotics have been shown to modulate the gut microbial ecosystem more efficiently than probiotics or prebiotics used in separate forms [91]. This enhanced efficacy stems from the distinct mechanisms of action of probiotics and prebiotics, which can modulate the gut microbiota in varied ways. The combination of these two approaches has been shown to promote a more balanced and diverse gut microbiota composition [91]. Consequently, there is a significant need for further investigation, through empirical research, to explore the potential benefits of multi-strain probiotics and synbiotics. Such studies will facilitate more profound analyses, offering enhanced insights into the mechanisms underlying these effects.

**Table 6:** Impact of the interaction between different strains of probiotics and synbiotics in aquaculture.

Country	Host × Probiotics	Experimental Condition	Main Outcomes	Reference
Bangladesh	▪ Climbing perch (*Anabas testudineus*). ▪ Pro-1 (*Lactobacillus* spp., minerals, fatty acid, amino acid, and chitosan), Pro-2 (*B. subtilis*, Lactic acid bacteria, and *S. cerevisiae*), Pro-3 (*B. subtilis*, *B. licheniformis*, *B. megaterium*, *L. acidophilus*, L. *plantarum*, S. *cerevisiae*), and Pro-4 (*Lactobacillus* spp., *B. subtilis*, *B. licheniformis*, *Aspergillus oryzae, A. niger*, *S. boulardii*, enzymes	▪ Dosage = 1 g/kg ▪ Experimental period: 8 weeks ▪ fish (~3.01 g) were distributed in 15 fish tanks (400 L capacity). ▪ Treatment 1 (T1) received diet supplemented with pro-1, while T2 to T4 received pro-2 to pro-4, respectively. ▪ Temperature = 26 ± 2.54 °C, photoperiod was 12 light/12 dark, pH = 7.4 ± 0.6, and DO = 6.74 ± 0.7 ppm. Fish samples were injected by *Aeromonas hydrophila* ▪ Supplementation: feed additive	▪ FBW (g): T1 ↑, T2 ↑, T3 ns, T4 ↑ ▪ FCR (g): T1 ↓, T2 ↓, T3 ↓, T4 ↓ ▪ SGR (% day^−1^): T1 ↑, T2 ↑, T3 ns ↑, T4 ↑ ▪ ALT and AST: T1 ↓, T2 ↓, T3 ↓, T4 ↓ ▪ WBC (10^9^/L): T1 ns, T2 ns, T3 ↑, T4 ns ▪ RBC (10^12^/L) T1 ↑, T2 ↑, T ns ↑, T4 ns ▪ Cumulative death (%) caused by *A. hydrophila* was ↑ in C followed by T1 and T2	[92]
Kazakhstan	▪ Nile tilapia (*Oreochromis niloticus*) ▪ *Leuconostoc mesenteroides* (LM), *L*. *lactis* (LL) and a combination of both probiotics (mix)	▪ Feeding trial: 8 weeks ▪ Fish (45 ± 2 g) were distributed in 4 groups (96 fish/group) with triplicate (32 fish/tank), tank size: 500 L. Group 1 received feed free probiotic, G2 to G4 received diet supplemented with LM, LL, and mix respectively. ▪ Supplementation: feed additive ▪ Temperature: 27 °C. ▪ Feeding rate: twice/day	▪ FBW (g): C: 68.4 ± 3.26 a, LM: 79.2 ± 1.16 b, LA: 78.5 ± 2.15 b, mix: 92.4 ± 4.54 c ▪ WG (%): C: 22.4 ± 3.01 a, LM: 31.7 ± 1.52 b, LA: 31.2 ± 1.74 b, mix: 47.3 ± 2.02 c ▪ SGR (%): C: 1.96 ± 0.11 a, LM: 2.32 ± 0.5 b, LA: 2.30 ± 0.3 b, mix: 2.82 ± 0.2 c	[93]
Australia	▪ Barramundi (*Lates calcarifer*) ▪ Seaweed) *Sargassum linearifolium*) and the probiotics *Lactobacillus casei* and *Saccharomyces cerevisiae*	▪ 90 Fish (4.25 ± 0.09 g) were distributed in 9 tanks (350 L) in 3 groups (G) with triplicate design (30 fish/tank). G1: control, G2: received *S. linearifolium* with probiotics, and G3: received *S. linearifolium* without probiotics ▪ Supplementation: feed additive ▪ Feeding rate: 3 time/day ▪ DO: 6.7 ± 0.64 mg/L, temperature: 27.9 ± 0.47 °C, ammonia: <0.25 mg/L. ▪ Duration: 56 days	▪ FBW (g): G1 ↓, G2 ↑, G3 ↓ ▪ SGR (%/day): G1 ↓, G2 ↑, G3 ↓ ▪ G2: improved mucosal barrier function ▪ G2: glutathione peroxidase ↑, total antioxidant ↑, hsp ↓, tnf-α ↓, occludin ↑, claudin1 ↑ and nrf ↑ ▪ Mortality caused by *Vibrio harveyi* was 27% in G2, 46% in G3, and 67% in G1.	[94]
China	▪ Hybrid groupers (*Epinephelus fuscoguttatus* ♀ *× E. lanceolatus* ♂) ▪ *Bacillus cereus* G1–11 and *Exiguobacterium acetylicum* G1–33	▪ 600 fish (53.30 ± 0.50 g) were distributed in 20 tanks (170 L). The experiment had 5 groups: probiotic free (C), *B. cereus* (BC), *E. acetylicum* (EA), mixed probiotic (mix), and positive control (P). ▪ Supplementation: feed additives ▪ Salinity: 24–30‰, temperature: 28 ± 2 °C ▪ Duration: 60 days	▪ WG (g): C ↓, P ↑, BC ↑, EA ↑, mix ↑ ▪ WGR (%): C ↓, P ↑, BC ↑, EA ↑, mix ↑. ▪ SGR (%/day): C ↓, P ↑, BC ↑, EA ↑, mix ▪ Amylase: C ↓, P ↑, BC ↑, EA ↑, mix ↑. ▪ Lipase: C ↓, P ↑, BC ↑, EA ↑, mix ↑ ▪ Pepsin: C ↓, P ↑, BC ↑, EA ↑, mix ↑ ▪ Growth and digestive enzymes, growth related gene expression were the highest in the mixed probiotic	[95]
Egypt	▪ *Mugil capito* ▪ *Saccharomyces cerevisiae*, *Lactobacillus bulgaricus*	▪ Fish (10.30 ± 0.10 g) were allocated into 4 groups (G) as the following: G1: Control diet, G2–G4: received diet supplemented with *S. cerevisiae* (4 g/kg diet), G3: *L. bulgaricus* (2 g/kg diet), G4: combination of both ▪ Supplementation: feed additives ▪ Duration: 60 days	▪ WG (g), WGR (%), SGR (%), FCR, PER were the best in all probiotic groups, however, G4: was significantly better than G3 and G2. ▪ Antioxidant enzymes ↑ in G4 than other groups.	[96]
Republic of Korea	▪ *Abalone (Haliotis discus hannai*) ▪ *Bacillus* sp. *KRF-7*, *Bacillus* sp. *PM8313*, *β-glucooligosaccharide*	▪ Abalone (1.32 ± 0.05 g) were fed diet supplemented with G1: control diet, G2: control diet + *Bacillus* sp. *KRF-7*, G3: *Bacillus* sp. *PM8313*, G3: *β glucooligosaccharide*, and G4: fed with their synbiotics. ▪ Duration: 90 days	▪ FBW (g): G1 ↓, G2 ↑, G3 ↑, G4 ↑ ▪ Lipase and amylase: G1 ↑, G2 ↑, G3 ↑, G4 ↑	[97]
Indonesia	▪ catfish (*Clarias gariepinus*) ▪ *Lactobacillus casei* and *Bacillus subtilis*.	▪ Catfish (80–100 g) were distributed into 4 groups. G1: received probiotic free diet, while G2–G4 received diet supplemented with 5, 10, and 15% probiotic respectively ▪ Supplementation: feed additive ▪ Duration: 42 days	▪ FBW (g): G1 ↓, G2 ↓, G3 ↓, G4 ↑ ▪ BWG (%): G1, G2 ↓, G3 ↑, G4 ↑ ▪ SGR (%/day): G1 ↓, G2 ↓, G3 ↓, G4 ↑ ▪ TNF-α: G1, G2 ↑, G3 ↑, G4 ↑ ▪ IL-1: G1, G2 ↑, G3 ↑, G4 ↑	[83]
Republic of Korea	▪ flounder (*Paralichthys olivaceus*) ▪ *Rummeliibacillus* sp. and *Microbacterium* sp.	▪ Fish (~11.4 g) were distributed into 2 groups. CON received probiotic diet while PRO received diet supplemented with a mixture of probiotics ▪ Supplementation: feed additive ▪ Feeding: 2 times/day ▪ Duration: 42 days	▪ FBW (g): CON ↓, PRO ↑ ▪ SGR (%/day): CON ↓, PRO ↑ ▪ FCR: CON ↑., PRO ↓ ▪ MPO: CON ↓, PRO ↑ ▪ phylogenetic diversity: CON ↓, PRO	[74]
Pakistan	▪ Tilapia (*Oreochromis niloticus*) ▪ *Lactobacillus plantarum* and *Pediococcus pentosaceus*	▪ Fish (6.13 ± 0.83) were distributed into 4 groups. G1: received probiotic free diet, while G2 ± G4 received diet supplemented with *L. plantarum*, *P. pentosaceus*, and mixture of both probiotics ▪ Supplementation: feed additive ▪ Duration: 60 days	▪ FBW (g): G1, G2 ↑, G3 ↑, G4 ↑ ▪ WGR (%): G1, G2 ↑, G3 ↑, G4 ▪ SGR (%/day): G1, G2 ↑, G3 ↑, G4 ↑ ▪ FCR: G1 ↑, G2, G3, G4 ▪ TL: G1, G2 ↑, G3 ↑, G4 ↑ ▪ HL: G1, G2 ↑, G3 ↑, G4 ↑ ▪ All the parameters were the best in probiotics mixture.	[98]
Pakistan	▪ Rohu (*Labeo rohita*) ▪ *Bacillus subtilis* and *Saccharomyces cerevisiae*	▪ Fish (11.00 ± 2.34 g) were distributed into 2 groups. G1 received control diet only while G2 received diet supplemented with a mixture of probiotics (1 × 10^6^ CFU g^−1^ *Bacillus subtilis* and 1 × 10^6^ CFU g^−1^ *Saccharomyces cerevisiae*) ▪ Duration: 3 months ▪ Supplementation: feed additive	▪ FBW (g): G1 ↓, G2 ↑ ▪ WG (g): G1 ↓, G2 ↑ ▪ WG% (g): G ↓ ↑, G2 ↑ ▪ FBB: G1 ↓, G2 ↑ ▪ FCR: G1 ↑, G2 ↓	[99]

↑: increase, ↓: decrease, Different letters mean significant differences. ns: non-significant differences (*p* < 0.05)

## 4. Key Limitations of Aquaculture Probiotics

The utilization of probiotics in aquaculture has received considerable attention as an alternative solution to the use of antibiotics to enhance fish performance. However, the use of probiotics in aquaculture has limitations despite its potential benefits. One of the main limitations of the use of probiotics in aquaculture is their specificity and variability across species and environmental conditions. Probiotics must be adapted to specific host species to achieve optimal results, as a bacterium that is beneficial to one species may not provide the same benefits to other hosts [100]. In addition, the bacteria that are beneficial to certain fish species may have a pathogenic effect on another fish species [100]. This species specification requires the extensive testing and characterization of probiotic strains before they can be effectively used in aquaculture [101]. In addition, the environmental conditions in which probiotics are applied, such as the water temperature, salinity, and pH, can significantly affect their survival, effectiveness, and stability [39,102]. For example, probiotics must be able to withstand the harsh conditions of the fish gastrointestinal tract, including exposure to bile salts and low pH [103]. In addition, some aquaculture probiotics have been associated with alterations in the dynamics of nutrient cycling. Although probiotics are frequently implemented to enhance nutrient utilization and promote water quality improvement, their presence can also result in unforeseen consequences. For instance, the introduction of particular probiotic strains has been observed to suppress the growth of cyanobacteria by increasing the relative abundance of other microbial groups, such as Verrucomicrobiota (from 2.61% to 6.35%) [104]. This alteration, while it seems beneficial, has the potential to disrupt the natural balance of primary producers in aquatic ecosystems, which may result in declines in biodiversity in aquatic ecosystems [104]. These factors may limit the number of probiotic strains that can be effectively used in aquaculture.

## 5. Conclusions

Probiotics have demonstrated efficacy in improving the growth performance, feed utilization, disease resistance, and water quality management in various aquaculture systems. The mechanisms underlying these effects, which include improving gut health, modulating immune responses, and reducing environmental stressors, position probiotics as an essential tool in promoting sustainable aquaculture. The present bibliometric study highlights the importance of probiotics as a viable solution within the context of aquaculture. A detailed analysis of global research trends reveals a substantial increase in the publication of APGD documents from 2019 to 2024, signifying a notable rise in scientific and practical interest in probiotics as eco-friendly alternatives to antibiotic use in aquaculture. Asia, particularly China, has emerged as a leader in research on the use of probiotics in aquaculture, driven by its substantial aquaculture industry. A bibliometric analysis using VOSviewer software also revealed a significant co-occurrence of keywords such as “disease resistance”, “growth performance”, and “gut microbiota”, underscoring their prominence in recent research efforts. This may indicate that the majority of APGD documents are focused on the relationship between disease resistance and probiotics.

According to the recently reviewed literature, probiotics such as *Pediococcus* spp., *Lactobacillus casei*, *Lactobacillus planta*, *Clostridium butyricum*, *Bacillus subtilis*, *Saccharomyces cereviciae*, and *Halobacterium salinarum* have shown remarkable improvements in a wide range of aquaculture species through many ways such as improving their growth parameters, feed utilization, and immune-related parameters. In addition, the synergistic effects of multi-strain probiotics and synbiotics have demonstrated promising results in optimizing health benefits, highlighting the need for further experimental investigation.

A well-conducted bibliometric review can play a powerful role in advancing the discipline of aquaculture probiotics by providing a comprehensive perspective, identifying the research gaps, and establishing guidelines for future research studies. Further research is needed to explore strain-specific effects, optimal dosages, timing, combinations of probiotics with prebiotics, and the impacts on aquatic ecosystems for the purpose of sustainable aquaculture. By incorporating probiotics into aquaculture practices, the industry can meet the growing demand for aquatic products while aligning with global sustainability goals.

## Figures and Tables

**Table 1:** The top 20 co-authoring countries of APGD documents based on WoS, 2008–2024.

Rank	Country	No. of Documents	% of 175	Citations	Link Strength
1	China	56	32.347	596	09
2	Iran	15	8.671	664	25
3	India	14	8.092	703	22
4	Malaysia	13	7.514	812	17
5	Spain	13	7.514	806	22
6	Thailand	13	7.514	513	24
7	Egypt	13	7.514	449	22
8	Bangladesh	12	6.936	190	14
9	USA	11	6.358	243	15
10	Norway	09	5.172	506	19
11	South Korea	09	5.172	311	12
12	Australia	08	4.598	474	15
14	Italy	06	3.448	253	12
15	Japan	06	3.448	78	08
16	Pakistan	06	3.448	24	06
17	Saudi Arabia	05	2.874	273	14
18	Mexico	04	2.299	44	02
19	Poland	04	2.299	18	06
20	Portugal	04	2.299	130	03

APGD: aquaculture, probiotics, growth performance, and disease resistance.

**Table 2:** Co-occurrence and total link strength of top keywords in the APGD documents based on WoS, 2008–2024.

Keywords	Occurrence	Total Link Strength
Disease resistance	130	1793
Probiotics	99	1348
Aquaculture	88	1169
Growth performance	67	913
Bacillus subtilis	35	491
Fish	36	482
Performance	34	456
Immunity	31	434
Trout (*Oncorhynchus mykiss*)	28	402
Dietary supplementation	28	396
Rainbow trout	27	385

**Table 3:** Co-occurrence and total link strength of the most powerful author keywords in APGD documents based on WoS literature searches, 2008–2024.

Keywords	Occurrence	Total Link Strength
Probiotics	62	291
Aquaculture	30	145
Immunity	24	122
Probiotic	22	109
Growth performance	21	108
Disease resistance	20	91
Growth	12	58
*Bacillus subtilis*	11	54
*Litopenaeus vannamei*	11	49
Gut microbiota	10	51
Immune response	08	39
Intestinal microbiota	08	38
Intestinal health	08	34
Gene expression	07	34
Feed additives	07	31
*Aeromonas hydrophila*	06	33
Microbiota	06	26

APGD: aquaculture, probiotics, growth performance, and disease resistance.

## Data Availability

Data supporting this study’s findings are available from the corresponding author upon request.
